# Gender difference in plasma fatty-acid-binding protein 4 levels in patients with chronic obstructive pulmonary disease

**DOI:** 10.1042/BSR20150281

**Published:** 2016-02-29

**Authors:** Xue Zhang, Diandian Li, Hao Wang, Caishuang Pang, Yanqiu Wu, Fuqiang Wen

**Affiliations:** * Department of Respiratory and Critical Care Medicine, West China Hospital of Sichuan University and Division of Pulmonary Diseases, State Key Laboratory of Biotherapy of China, Chengdu 610041, China

**Keywords:** chronic obstructive pulmonary disease, fatty-acid-binding protein 4, inflammation, lung function

## Abstract

COPD (chronic obstructive pulmonary disease) is characterized by airway inflammation and increases the likelihood of the development of atherosclerosis. Recent studies have indicated that FABP4 (fatty-acid-binding protein 4), an intracellular lipid chaperone of low molecular mass, plays an important role in the regulation of inflammation and atherosclerosis. We carried out a preliminary clinical study aiming at investigating the relationships between circulating FABP4 levels in patients with COPD and inflammation and lung function. We enrolled 50 COPD patients and 39 healthy controls in the study. Lung function tests were performed in all subjects. Plasma levels of FABP4 and adiponectin, TNFα (tumour necrosis factor α) and CRP (C-reactive protein) were measured. The correlations between FABP4 and lung function, adipokine (adiponectin), inflammatory factors and BMI (body mass index) were analysed. Compared with both males with COPD and healthy females, plasma FABP4 levels in females with COPD were significantly increased. Adiponectin and CRP levels were significantly higher in patients with COPD. Furthermore, we found that FABP4 levels were inversely correlated with FEV_1_% predicted (FEV_1_ is forced expiratory volume in 1 s) and positively correlated with adiponectin and TNFα in COPD patients. In addition, a positive correlation between plasma FABP4 and CRP was found in females with COPD. However, FABP4 levels were not correlated with BMI. Our results underline a gender difference in FABP4 secretion in stable COPD patients. Further studies are warranted to clarify the exact role of FABP4 in the pathogenesis of COPD.

## Abstract (precis)

Plasma FABP4 levels were higher in females with COPD compared with both males with COPD and healthy females. FABP4 levels correlated inversely with lung function, and positively with adiponectin and TNFα in COPD.

## INTRODUCTION

COPD (chronic obstructive pulmonary disease) is characterized by persistent and usually progressive airflow limitation [1]. With a high prevalence of 10.2%, COPD is a leading cause of mobility and mortality worldwide, and is becoming an economic and social burden [1,2]. Amplifying inflammatory reactions to noxious stimuli is recognized as an important pathogenesis of COPD, which involve numerous inflammatory factors and various immune cells [3,4]. Besides the abnormalities in the airway, many COPD patients are affected by co-morbidities, such as metabolic disorders, cardiovascular diseases and osteoporosis, which have a strong impact on the disease prognosis and quality of life [1]. According to the present-day view, systemic inflammation is the key between pulmonary disease and the systemic co-morbidities [5].

Adipocytes produce and secrete various bioactive peptides, known as adipokines. Adiponectin, an anti-inflammatory adipokine, is regarded as a candidate biomarker of COPD [6,7]. FABP4 (fatty-acid-binding protein 4), a member of the FABP family, has recently been suggested as another adipokine [8]. FABP4 is produced in adipocytes, macrophages and endothelial cells [8,9]. Numerous studies have elucidated the roles of FABP4 in body-weight control, glucose and lipid metabolism, β-cell function and the pathogenesis of atherosclerosis [9–11]. Recent studies suggest that FABP4 is involved in the regulation of macrophages and airway inflammation [12–14]. FABP4-deficient mice showed protection from atherosclerosis and FABP4-deficient macrophages influenced the production of inflammatory cytokines [15]. In a model of allergic airway inflammation *in vivo*, FABP4 participated in the infiltration of leucocytes into the airways [14]. In addition to the cytoplasm, FABP4 can also be released into the circulation. Elevated levels of FABP4 in the circulation were found in many diseases, such as bronchopulmonary dysplasia, SLE (systemic lupus erythaematosus) patients and OSA (obstructive sleep apnoea) [13,16,17].

Therefore, since COPD is a disease with inflammation and high risk of atherosclerosis [18,19], plasma FABP4 levels may be closely related to COPD. However, at present, we are unaware of any published studies investigating the relationship between circulating FABP4 levels and COPD. We therefore conducted the present study to fill the research gap, trying to find a possible association of plasma FABP4 and COPD.

## MATERIALS AND METHODS

### Subjects

The protocol of the study was approved by the Institutional Review Board for Human Studies of West China Hospital of Sichuan University (China) and accords with the principles of the Declaration of Helsinki. All subjects provided written informed consent. The study was conducted from March 2013 to February 2014. Patients with COPD were enrolled from the Outpatient Department of West China Hospital and healthy controls were enrolled from the Hospital's Physical Examination Centre. According to the GOLD (Global Initiative for Obstructive Lung Disease) guidelines [1], patients enrolled in the study should meet the following criteria: FEV_1_ (forced expiratory volume in 1 s)/FVC (forced vital capacity) less than 70% after inhalation of bronchodilator and increase of FEV_1_ after inhalation of bronchodilator less than 12%. Exclusion criteria included acute exacerbation of COPD in the 3 months before the study, respiratory disorders (asthma, pulmonary fibrosis, lung cancer, bronchiectasis and tuberculosis) and diseases known to be associated with elevated levels of circulating FABP4 (metabolic and vascular disease), according to the clinical history. No patients had taken glucocorticoids or bronchodilators previously.

### Analysis of plasma FABP4, adiponectin, TNFα and CRP

Subjects were asked not to eat anything in the morning until blood was taken. Venous blood was drawn from all subjects and plasma was collected. After centrifugation at 400 ***g*** for 10 min at 4°C, the samples were stored at −80°C until analysis. Plasma levels of FABP4 were analysed using a commercial ELISA kit (Neobioscience) and adiponectin, TNFα (tumour necrosis factor α) and CRP (C-reactive protein) levels were performed using a Luminex Bio-Plex 200 system (Bio-Rad Laboratories) according to the manufacturer's protocols. The limitations of detection are 0.8 ng/ml for FABP4, 148 pg/ml for adiponectin, 1.2 pg/ml for TNFα and 116 pg/ml for CRP. Other blood parameters, such as TAG (triacylglycerol), TC (total cholesterol), HDL (high-density lipoprotein), LDL (low-density lipoprotein) and glucose, were examined by the Department of Laboratory Medicine of West China Hospital. Clinical data, such as age, pack-years of smoking and blood pressure, were collected. All analyses were performed by technicians who were blind to the characteristics of the subjects.

### Statistics

All data were analysed using SPSS (version 19.0, SPSS Inc.). Results are presented as means±S.D. For comparison of continuous variables between two groups, unpaired Student's *t* test was used after confirming data met normal distribution. For parameters with skewed distributions, logarithmic transformation was performed before further analysis. For comparing multiple groups, one-way ANOVA was carried out. A χ^2^ test was used for comparison of categorical variables. Correlations were analysed using a bivariate Pearson's correlation test. The correlation between FABP4 and other variables was evaluated using a multivariate linear regression model. *P*<0.05 was considered to be statistically significant.

## RESULTS

### Study subjects

The study enrolled 50 COPD patients and 39 healthy controls in total. The characteristics of COPD patients are shown in Table 1. Most patients had moderate COPD, according to the GOLD guidelines [1]. No differences were found in age and gender between male and female COPD patients. Other parameters, such as BMI, blood pressure, metabolism parameters and FEV_1_% predicted were also similar between groups.

**Table 1:** Baseline characteristics of COPD patients and healthy controls SaO_2_, oxygen saturation. *P<0.01 compared with healthy controls, †*P*<0.01 compared with female counterparts.

	COPD			Controls		
Characteristic	Total (*n*=50)	Male (*n*=30)	Female (*n*=20)	Total (*n*=39)	Male (*n*=23)	Female (*n*=16)
Age (year)	64.14±10.21	66.37±9.05	60.80±11.15	60.92±9.62	60.78±10.52	61.13±8.48
BMI (kg/m^2^)	22.56±2.60	22.38±3.03	22.81±1.83	23.59±3.61	24.05±3.42	22.92±3.87
Systolic blood pressure (mmHg)	126.36±17.56	127.80±19.01	124.20±15.34	123.62±17.87	125.39±17.95	121.06±18.02
Diastolic blood pressure (mmHg)	76.04±9.47	74.90±9.17	77.75±9.89	76.97±11.57	77.09±12.75	76.81±10.05
TAG (mmol/l)	1.76±0.72	1.63±0.67	1.96±0.76	1.49±0.65	1.45±0.63	1.54±0.71
TC (mmol/l)	5.10±0.64	4.99±0.74	5.28±0.43	4.80±0.85	4.74±0.84	4.88±0.90
HDL (mmol/l)	1.99±0.50	2.05±0.51	1.90±0.50	1.83±0.67	1.91±0.78	1.72±0.48
LDL (mmol/l)	2.29±0.67	2.27±0.76	2.31±0.51	2.14±0.80	2.08±0.81	2.23±0.82
Glucose (mmol/l)	5.39±0.91	5.33±1.07	5.48±0.62	5.26±1.31	5.44±1.57	5.00±0.76
SaO_2_ (%)	96.18±2.07	96.23±2.13	96.10±2.02	96.77±1.60	96.48±1.81	97.19±1.67
Smoking (pack-years)	14.29±20.69	22.82±22.49†	1.50±6.71	9.29±14.85	15.75±16.57†	0
FEV_1_% predicted	62.86±12.77*	62.93±11.92	62.75±14.27	111.67±17.86	110.04±18.71	114.00±16.87

### Plasma levels of FABP4, adiponectin and inflammatory biomarkers

**Figure 1:**
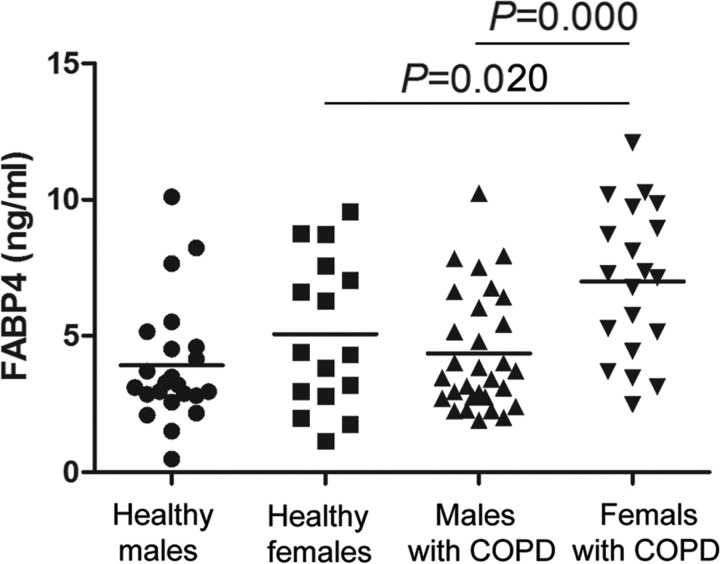
Plasma levels of FABP4 in the healthy males group, healthy females group, males with COPD group and females with COPD group The horizontal line represents the median value and one-way ANOVA was used to determine significance. *P*<0.05 was considered to be statistically significant.

Compared with healthy subjects, no significant differences were found in the levels of FABP4 in the COPD patients. When comparing males and females, females had significantly higher levels of FABP4 than males in the COPD group (6.66±2.85 ng/ml compared with 4.35±2.16 ng/ml, *P*<0.0001; Figure 1). However, there was no significant difference between males and females in the control group (3.91±2.22 ng/ml compared with 5.06±2.74 ng/ml, *P*=0.157; Figure 1). When subjects were stratified for gender, FABP4 levels in COPD group were significantly higher than those in control group for females (*P*=0.020) and no significance was found for males (*P*=0.515) (Figure 1). In addition, compared with controls, significantly higher levels of adiponectin (7.81±4.37 μg/ml compared with 5.64±2.69 μg/ml, *P*=0.005) and CRP (2.93±3.32 μg/ml compared with 0.97±1.08 μg/ml, *P*<0.0001) were found in COPD patients (Table 2). There was no difference in plasma TNFα levels between the two groups (Table 2).

**Table 2:** Plasma concentration of FABP4, adiponectin and inflammatory biomarkers of COPD and controls #Logarithmic transformation values were used for statistical analyses of parameters with skewed distributions, *P<0.05, **P<0.01 compared with healthy controls, †*P*<0.05, ‡*P*<0.01 compared with female counterparts.

	COPD			Controls		
	Total (*n*=50)	Male (*n*=30)	Female (*n*=20)	Total (*n*=39)	Male (*n*=23)	Female (*n*=16)
FABP4 (ng/ml)	5.27±2.69	4.35±2.16‡	6.66±2.85*	4.38±2.48	3.91±2.22	5.06±2.74
Adiponectin (μg/ml)#	7.81±4.37**	7.78±4.19	7.85±4.75	5.64±2.69	4.78±2.03†	6.88±3.08
TNFα (pg/ml)#	16.05±20.39	12.79±19.65	20.94±21.00	14.77±22.07	17.36±27.14	11.06±11.35
CRP (μg/ml)#	2.93±3.32**	3.35±3.66	2.29±2.72	0.97±1.08	0.84±1.05	1.15±1.14

### Correlation analysis

**Figure 2:**
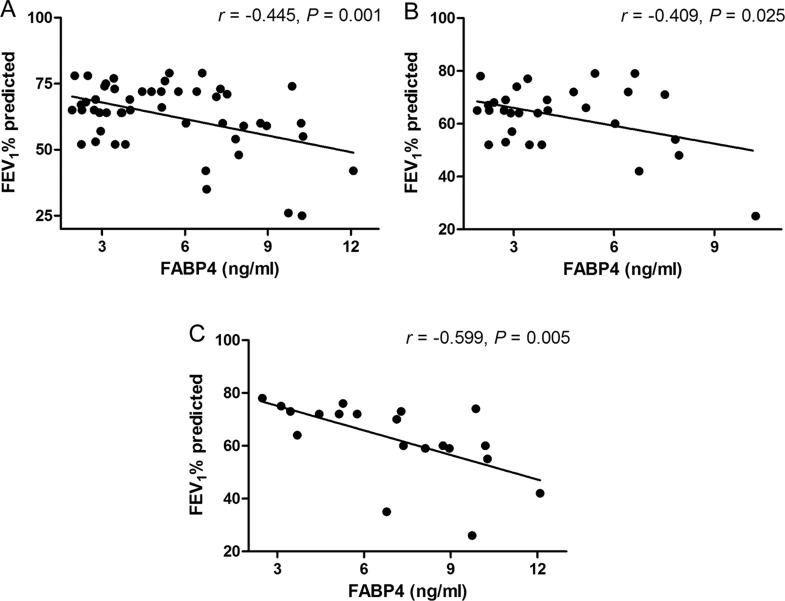
Correlation analysis between plasma levels of FABP4 and lung function (FEV_1_% predicted) in COPD patients (A), males with COPD (B) and females with COPD (C) Pearson's correlation test was used and the correlation coefficient is presented as an *r* value.

For patients with COPD, the levels of plasma FABP4 were inversely correlated with FEV_1_% predicted (*r*=−0.445, *P*=0.001; Figure 2A). As shown in Table 3, plasma levels of FABP4 were positively correlated with plasma TNFα (*r*=0.327, *P*= 0.020) and adiponectin (*r*=0.283, *P*=0.046) in the COPD group. When patients were stratified for gender, inverse correlations were found between FABP4 levels and FEV_1_% predicted for males (*r*=−0.409, *P*=0.025; Figure 2B) and for females (*r* =−0.599, *P*=0.005; Figure 2C) and levels of CRP in plasma were positively correlated with FABP4 levels for females (*r*=0.511, *P*=0.021; Table 3). We also analysed the correlation between BMI and FABP4 levels and no correlation was found in each group. In addition, we examined the association of FABP4 with various parameters in COPD patients using multivariate linear regression analysis. This analysis demonstrated that plasma FABP4 levels were independently associated with gender and FEV_1_% predicted (Table 4). However, no correlation was found between any parameter and FABP4 levels in the control group.

**Table 3:** Correlations between FABP4 and adiponectin and inflammatory biomarkers in COPD patients The bold values indicate significant differences (*P*<0.05).

	Total (*n*=50)		Male (*n*=30)		Female (*n*=20)	
	*r*	*P-*value	*r*	*P-*value	*r*	*P-*value
Adiponectin	0.283	**0.046**	0.288	0.123	0.352	0.128
TNFα	0.327	**0.020**	0.292	0.118	0.248	0.292
CRP	0.016	0.914	−0.141	0.457	0.511	**0.021**

**Table 4:** Multivariable linear regression analysis for plasma FABP4 in COPD patients The bold values indicate significant differences (*P*<0.05).

Parameter	Standardized β coefficient	*P*-value
Age	−0.058	0.635
Gender	0.283	**0.049**
BMI	0.027	0.832
Smoking (pack-years)	−0.162	0.242
Adiponectin	0.158	0.250
TNFα	0.227	0.067
CRP	−0.080	0.511
FEV_1_% predicted	−0.398	**0.004**

## DISCUSSION

To the best of our knowledge, the present study is the first to examine the plasma levels of FABP4 in COPD patients and the correlations between FABP4 and adiponectin, inflammatory biomarkers and lung function. The present study suggests that FABP4 levels were significantly higher in females with COPD compared with both males with COPD and healthy females. Furthermore, FABP4 levels were correlated with lung function, which is measured as FEV_1_% predicted in the COPD group. We also found that levels of FABP4 were positively correlated with TNFα and adiponectin in the COPD group. In females with COPD, FABP4 levels were positively correlated with CRP.

Circulating FABP4 has been demonstrated to participate in the pathogenesis of atherosclerosis and to be closely related to insulin resistance, Type 2 diabetes and the metabolic syndrome [9,10]. We found in the present study that the levels of FABP4 were higher in COPD patients than in healthy female controls, indicating that FABP4 may play a role in COPD, especially for females. Macrophages induced by cigarette smoke and other irritants can release inflammatory factors in COPD [20]. FABP4 is involved in the activation of transcription factors and release of inflammatory factors in macrophages which may play a role in the pathogenesis of COPD. FABP4-deficient macrophages display reduced IκB [inhibitor of NF-κB (nuclear factor κB)] kinase and NF-κB activity in pro-inflammatory responsiveness, resulting in diminished inflammatory function and decreased inflammatory cytokines, such as TNFα, IL-6 (interleukin 6) and MCP-1 (monocyte chemoattractant protein 1) [21]. COPD is characterized by chronic inflammation in the airway [22] and FABP4-knockout mice display decreased airway inflammation in response to VEGF (vascular endothelial growth factor) [23]. Our previous study has shown that serum levels of oxLDL (oxidized LDL) are increased and associated with CRP and ROS (reactive oxygen species) levels in COPD patients [24] and oxLDL can induce the production of FABP4 [25,26]. In addition, we also found that FABP4 levels were associated with FEV_1_% predicted in COPD patients which suggests FABP4 may be correlated with airway obstruction.

Our results present the differences between males and females. We compared BMI, which is a potential influence factor between the two groups, and no significant differences were found. The reason for this phenomenon remains unknown. As mentioned in a previous study, a difference in regional fat distribution and sex hormones may contribute to the gender difference [27]. Generally, men have more abdominal (visceral) fat, and females have more subcutaneous fat. Compared with omental adipose tissue, the expression of FABP4 was higher in subcutaneous fat in obese subjects [28]. Females usually have a greater amount of body fat than males and this may be another reason, since there is a strong and independent association of FABP4 levels and adiposity [29]. Furthermore, FABP4 levels are found to be negatively correlated with free testosterone in women [30]. It is therefore tempting to speculate that testosterone suppresses the expression of FABP4, which may explain the gender difference. More studies are needed to unravel this phenomenon.

COPD patients are more likely to develop metabolic disorders and cardiovascular diseases [31]. Accumulating data provide evidence that FABP4 plays a role in the metabolic syndrome, insulin resistance and atherosclerosis [8]. For example, in contrast with control mice, FABP4-deficient mice did not develop insulin resistance or diabetes in diet-induced obesity mode [32] and *Fabp4* mRNA expression in circulating leucocytes isolated from *Apoe*^−/−^ (apolipoprotein E-null) mice was correlated with atherosclerotic lesion size [33]. We therefore speculate that the elevated levels of FABP4 in COPD patients, especially in females, may link COPD with metabolic and cardiovascular disorders.

Plasma levels of adiponectin, which is also an adipokine, were elevated in COPD group in the present study. Adiponectin, mainly studied in metabolic diseases, has anti-inflammatory, anti-atherogenic and anti-diabetic effects [34]. The results of studies of adiponectin levels in COPD patients are inconclusive. The present study found that the levels of adiponectin in plasma were higher in the COPD group which is consistent with previous results [7]. A positive correlation between adiponectin and FABP4 was found. One explanation may be that both FABP4 and adiponectin can be produced and released from adipocytes [9] and are related to inflammation and metabolic disorders [8,34]. The present study also showed that plasma levels of CRP were higher in patients with COPD than healthy controls, which is consistent with previous findings [24], whereas TNFα levels were not different between the two groups. BMI in the two groups were similar, and this may contribute to the similar TNFα levels according to a previous study indicating that TNFα levels were constant in weight-stable patients [35]. In addition, we found FABP4 levels to be positively correlated with TNFα in the COPD group and CRP in female patients. TNFα and CRP are inflammatory factors and associated with COPD and FEV_1_ decline [36,37]. The correlation suggests that FABP4 may serve as a biomarker of systemic inflammation in COPD, especially for females, and systemic inflammation may be the cause of the link between elevated FABP4 levels and lung function impairment. To the best of our knowledge, no studies have investigated the effect of gender difference in FABP4 levels in patients with COPD. Hence we can only speculate about the possible effects of an increased FABP4 levels. After gender stratification, we found no significant correlation between FABP4 levels and adiponectin or TNFα. We speculate that gender might not be the main factor that influences the correlation analyses. This is in agreement with a previous study showing that there was no association between FABP4 and adiponectin or TNFα receptor 2 in morbidly obese women [38]. Moreover, the fact that the sample size was small when the groups were stratified may explain the non-significant correlation. Previously, FABP4 was demonstrated to play a role in regulating pro-inflammatory responses [21]. Since we observed gender differences in the present study, it is tempting to speculate that higher FABP4 in females may favour CRP production, and important differences may exist in adipocyte function between genders. Similarly, Bagheri et al. [39] found that circulating FABP4 was correlated with CRP concentration in females with Type 2 diabetes mellitus, but not in males. However, more studies are needed to verify the hypothesis.

Although we have obtained results from our preliminary study, some limitations should be taken into account when analysing the data. First, the number of subjects was small, especially when stratifying for gender. Given that obesity influences the levels of FABP4, our small sample size cannot conclude whether obesity contributes to the results. So more studies of large sample sizes are needed to clarify further the role of FABP4 in COPD. Secondly, most patients included had moderate COPD, so more investigations are necessary to evaluate further the levels of FABP4 in more severe COPD.

In conclusion, our results underline the gender-related difference of FABP4 secretion in COPD. Plasma levels of FABP4 were higher in females with COPD compared with both males with COPD and healthy females. Furthermore, FABP4 levels were correlated inversely with lung function and positively with adiponectin and TNFα in the COPD group. In females with COPD, the FABP4 concentration was positively correlated with CRP. Large-scale studies are needed to confirm our findings and further studies are required to clarify the impact of FABP4 on the pathogenesis of COPD.

## Abbreviations

COPD: chronic obstructive pulmonary disease
CRP: C-reactive protein
FABP: fatty-acid-binding protein
FEV_1_: forced expiratory volume in 1 s
GOLD: Global Initiative for Obstructive Lung Disease
HDL: high-density lipoprotein
LDL: low-density lipoprotein
NF-κB: nuclear factor κB
oxLDL: oxidized LDL
TC: total cholesterol
TAG: triacylglycerol
TNFα: tumour necrosis factor α
